# SARS-CoV-2 Accessory Protein ORF7b Mediates Tumor Necrosis Factor-α-Induced Apoptosis in Cells

**DOI:** 10.3389/fmicb.2021.654709

**Published:** 2021-08-13

**Authors:** Ruiping Yang, Qiong Zhao, Jingjing Rao, Feng Zeng, Shengren Yuan, Manshan Ji, Xiaoguang Sun, Jian Li, Jing Yang, Jingwen Cui, Zhixiong Jin, Long Liu, Zhixin Liu

**Affiliations:** ^1^School of Basic Medical Sciences, Hubei University of Medicine, Shiyan, China; ^2^Biomedical Research Institute, Hubei University of Medicine, Shiyan, China; ^3^Hubei Key Laboratory of Embryonic Stem Cell Research, Hubei University of Medicine, Shiyan, China

**Keywords:** SARS-CoV-2, ORF7b, apoptosis, interferon, immune responses

## Abstract

The accessory proteins of coronaviruses are essential for virus–host interactions and the modulation of host immune responses. It has been reported that accessory protein ORF3a encoded by severe acute respiratory syndrome coronavirus 2 (SARS-CoV-2) can induce apoptosis, and accessory protein ORF6 and ORF8 could be inhibitors of the type-I interferon (IFN) signaling pathway. However, the function of accessory protein ORF7b is largely unknown. We investigated the apoptosis-inducing activity of ORF7b in cells. Cytokine levels and host innate immune responses, including expression of interferon regulatory transcription factor (IRF)-3, signal transducer and activator of transcription (STAT)-1, interferon (IFN)-β, tumor necrosis factor (TNF)-α, and interleukin (IL)-6, were also investigated. We found that ORF7b promoted expression of IFN-β, TNF-α, and IL-6, activated type-I IFN signaling through IRF3 phosphorylation, and activated TNFα-induced apoptosis in HEK293T cells and Vero E6 cells. These results could provide deeper understanding about the pathogenicity of SARS-CoV-2 as well as the interaction between the accessory protein ORF7b with host immune responses.

## Introduction

Severe acute respiratory syndrome coronavirus 2 (SARS-CoV-2) is a β-coronavirus within the family *Coronaviridae* that infects humans. SARS-CoV-2 infection was discovered in many patients in Wuhan (Hubei Province, China) in December 2019. This infection was found to cause coronavirus disease 2019 (COVID-19; Zhu et al., 2020).

A pandemic of COVID-19 has spread rapidly worldwide since January 2020. As of June 12, 2021, >175 million cases have been confirmed and 3.78 million deaths documented (CSSE, 2021). COVID-19 has wreaked havoc on healthcare and economic systems worldwide.

In addition, increased levels of cytokines (e.g., IL-6, IL-10, and TNF-α) have been associated with severe COVID-19 (Chen et al., 2020). Compared with non-intensive care unit (ICU) patients, ICU patients have been reported to have higher plasma levels of IL-2, IL-7, IL-10, granulocyte colony-stimulating factor, interferon-gamma-inducible protein (IP)-10, monocyte chemotactic protein-1, MIP1A, and tumor necrosis factor (TNF)-α (Huang et al., 2020). In a way, the damage caused by SARS-CoV-2 infection is due mainly to a “cytokine storm” (Wang et al., 2020).

SARS-CoV-2 codes a variable number of accessory proteins which function in virus–host interactions and the modulation of host immune responses. These proteins may contribute to the pathogenicity of SARS-CoV-2 *via* different strategies (Narayanan et al., 2008; Liu et al., 2014). Scholars have reported that a SARS-CoV-2-encoded accessory protein called ORF3a can induce apoptosis *in vitro* (Ren et al., 2020). It has been found that the viral accessory proteins ORF6 and ORF8 could be potential inhibitors of the type-I interferon (IFN) signaling pathway (Li et al., 2020). However, the functions of a SARS-CoV-2-encoded accessory protein called ORF7b are not understood.

We examined the apoptosis-inducing activity of ORF7b from SARS-CoV-2 *in vitro*. Cytokine levels and host innate immune responses were also investigated. We believe that our results can provide a better understanding of ORF7b with the immune response and cytokine storm in the host.

## Materials and Methods

### Cell Culture and Transient Transfection

HEK293T cells and Vero E6 cells were cultured in the Dulbecco’s modified Eagle’s medium containing 10% fetal bovine serum (Gibco, Billings, MT, United States) in an atmosphere of 5% CO_2_ at 37°C. Cells were divided into 12 wells overnight before transient transfection. The plasmid (500 ng) encoding flag-ORF7b was transfected into cells for 18 h using Lipofectamine^™^ 3000 according to the manufacturer’s (Thermo Scientific, Waltham, MA, United States) instructions.

### Construction of Plasmids

*Orf7b* was amplified by polymerase chain reaction (PCR) using the forward primer 5'-TGGCCTCGAGATGATTGAACTTTCATTAATTG-3' and reverse primer 5'-TAATCCGCGGGGCGTGACAAGTTTCATTATG-3'. Then, fragments were digested by the restriction endonucleases *Xho*I and *Sac*II. The purified fragment was inserted into the pCAGGS-flag vector (pCAG-flag). The recombinant plasmid was screened by PCR and confirmed by sequencing and was called pCAG-flag-ORF7b or pCAG-flag-7b.

### Western Blotting

Whole cells were lysed and proteins quantified using a bicinchoninic acid kit (Beyotime, Beijing, China). Protein bands were separated by sodium dodecyl sulfate-polyacrylamide gel electrophoresis and transferred to polyvinylidene fluoride (PVDF) membranes. Following blockade by 5% skimmed milk, primary antibodies were incubated overnight at 4°C. After washing thrice, they were probed with horseradish peroxidase-coupled secondary antibodies. Finally, PVDF membranes were incubated with electrochemiluminescence reagent and signals were collected by Image Lab (Bio-Rad Laboratories, Hercules, CA, United States). The primary antibodies we used were monoclonal anti-FLAG^®^ M2 (catalog number, F1804; Sigma–Aldrich, Saint Louis, MO, United States), anti-signal transducer and activator of transcription (STAT)1 (ab234400; Abcam, Cambridge, United Kingdom), anti-phosphorylated (p) STAT1 (phospho S727; ab109461; Abcam), anti-interferon regulatory transcription factor (IRF)3 (ab68481; Abcam), anti-pIRF3 (phospho S386; ab76493; Abcam), anti-IL6 (66146-1-Ig; ProteinTech, Rosemont, IL, United States), anti-TNFα (60291-1-Ig; ProteinTech), and anti-glyceraldehyde 3-phosphate dehydrogenase (GAPDH; 60004-1-Ig; ProteinTech).

### Extraction of Total RNA and Real-Time Reverse Transcription-Quantitative Polymerase Chain Reaction

Total RNA was extracted using TRIzol^®^ Reagent according to the manufacturer’s (Invitrogen, Carlsbad, CA, United States) instructions. Complementary DNA was synthesized using the PrimeScript^™^ RT Reagent kit (TaKaRa Biotechnology, Shiga, Japan). Reverse transcription-quantitative polymerase chain reaction (QRT-PCR) was done using EvaGreen^®^ qPCR MasterMix (Applied Biological Materials, Richmond, Canada) in a CFX96 Real-Time PCR Detection System (Bio-Rad Laboratories). The primer pairs are listed in Supplementary Table S1. The thermocycling conditions were 95°C for 30 s followed by 40 cycles at 95°C for 5 s and 60°C for 30 s. GAPDH was used as the internal reference. Each reaction was set up in triplicate, and experiments were carried out thrice. Data were normalized to GAPDH expression using the 2^−ΔΔCt^ method (Livak and Schmittgen, 2001).

### Apoptosis Assay

Apoptosis of cells transfected with plasmids encoding flag-ORF7b or treated with inhibitors was detected using the Annexin V-fluorescein isothiocyanate (FITC) Apoptosis Staining/Detection kit (BMS500FI-300; eBioscience, San Diego, CA, United States). Cells (1 × 10^5^) from each well were collected 18 h after transfection, and 500 μl of binding buffer was added to resuspend them after washing thrice with phosphate-buffered saline. Then, cells were mixed with 5 μl of Annexin V-FITC and 2 μl of PI (Propidium Iodide, PI). Then, the cells incubated in the dark for 10 min at room temperature. Cell apoptosis was detected by flow cytometry using a CytoFLEX^™^ system (Beckman Coulter, Fullerton, CA, United States) and the experiment was repeated independently thrice.

### Detection of Caspase Activity and Enzyme-Linked Immunosorbent Assay

Caspase activity was measured using a fluorometric Multiplex Activity Assay Kit for caspase 3, caspase 8, and caspase 9 according to the manufacturer’s (ab219915, Abcam) instructions. ELISA kits for human IL-6 (KE00007; ProteinTech), TNF-α (KE00068; ProteinTech), and IFN-β (KE00187; ProteinTech) were used to detect cytokines in cell-culture supernatants according to the manufacturer’s instructions.

### Protein Extraction From the Nucleus and Cytoplasm, and Inhibitors

Proteins in the nucleus and cytoplasm were extracted using NE-PER^™^ Nuclear and Cytoplasmic Extraction Reagents (78835; Thermo Scientific) according to the manufacturer’s instructions. Z-IETD-FMK, a caspase-8 inhibitor (ab141382; Abcam) was dissolved in DMSO and was used at 50 μm unless stated otherwise. An antagonist of TNF-α receptors called R-7050 (5432; R&D Systems) was dissolved in DMSO and was used at 30 μm unless stated otherwise. An inhibitor of TNF-α called Infliximab (Y0002047; Sigma-Aldrich) was dissolved in PBS was used at 50 ng/ml unless stated otherwise. Human Serum Albumin (HSA; SRP6182; Sigma-Aldrich) was employed as control at 50 ng/ml.

### Data Analyses

Statistical analyses and data visualization were done using Prism 5.0 (GraphPad, San Diego, CA, United States). Data are the mean ± SD from three independent experiments. The significance of difference in values between groups was analyzed using the Student’s *t*-test or one-way ANOVA. The value of *p* < 0.05 was considered significant.

## Results

### ORF7b Induces Cell Apoptosis

**Figure 1:**
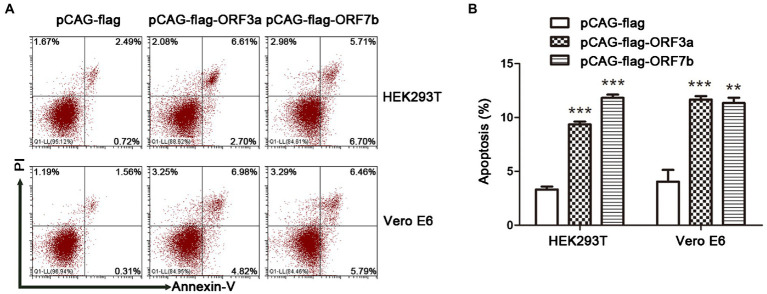
ORF7b induces apoptosis in HEK293T and Vero E6 cells. **(A)** Apoptosis was measured using flow cytometry at 18 h after transfection with pCAG-flag-ORF7b, pCAG-flag, and pCAG-flag-ORF3a groups were set as the negative control and positive control, respectively. **(B)** The percentage of cell apoptosis was quantified from three independent measurement. ^**^p < 0.01; ^***^p < 0.001, compared with the negative control group (one-way ANOVA and Tukey’s *post-hoc* test).

The accessory proteins encoded by SARS-CoV-2 have crucial roles in modulation of the host immune response and contribute to the pathogenicity of SARS-CoV-2. We studied whether accessory protein ORF7b could induce apoptosis. Because ORF3a has been proved to induce apoptosis in cells (Ren et al., 2020). Therefore, we detected the apoptosis of cells transfected with plasmids (pCAG-flag, pCAG-flag-ORF7b, and pCAG-flag-ORF3a), in which pCAG-flag was employed as negative control and pCAG-flag-ORF3a was employed as positive control. In our results, overexpression of ORF7b-flag or ORF3a-flag significantly increased the proportion of Annexin V-positive cells compared with control cells (Figure 1A). These results indicated that ORF7b could induce apoptosis in HEK293T cells and Vero E6 cells (Figure 1B).

### ORF7b Increases Expression of TNF-α, IL-6, and IFN-β

**Figure 2:**
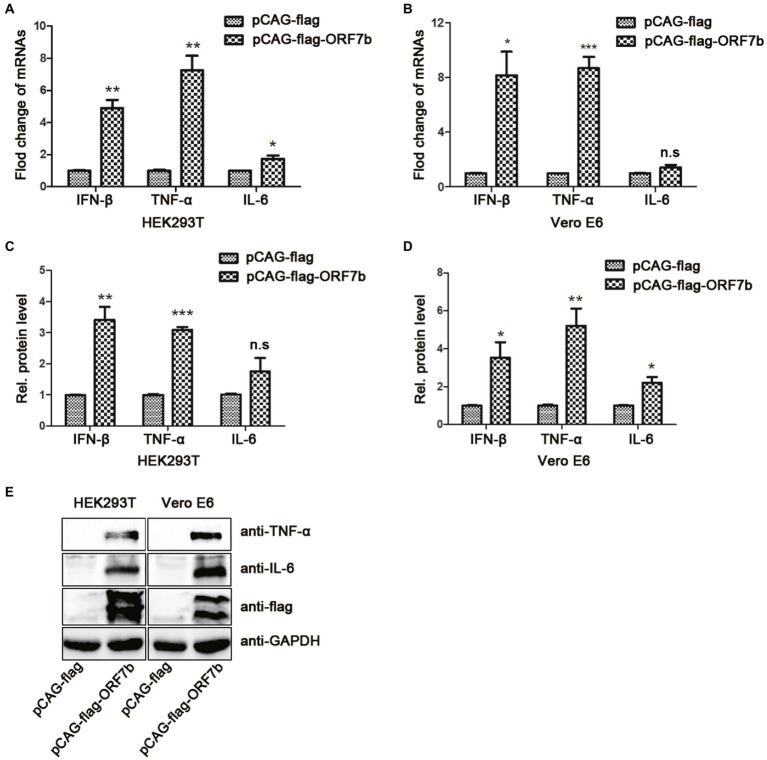
ORF7b increases the expression of tumor necrosis factor (TNF)-α, IL-6, and IFN-β. **(A)** mRNA levels of IFN-β, TNF-α, and IL-6 in HEK193T cells were determined by reverse transcription-quantitative polymerase chain reaction (QRT-PCR) at 18 h after transfection with pCAG-flag and pCAG-flag-ORF7b. **(B)** mRNA levels of IFN-β, TNF-α, and IL-6 in Vero E6 cells were determined by QRT-PCR at 18 h after transfection with pCAG-flag and pCAG-flag-ORF7b. **(C)** Expression of IFN-β, TNF-α, and IL-6 in HEK193T cell-culture supernatants was determined by ELISA at 18 h after transfection with pCAG-flag and pCAG-flag-ORF7b. **(D)** Expression of IFN-β, TNF-α, and IL-6 in Vero E6 cell-culture supernatants was determined by ELISA at 18 h after transfection with pCAG-flag and pCAG-flag-ORF7b. **(E)** Protein expression of TNF-α and IL-6 in Vero E6 cells and HEK293T cells was detected by Western blotting at 18 h after transfection with pCAG-flag and pCAG-flag-ORF7b. Data are the mean ± SD, *n* = 3. ^*^p < 0.05, ^**^p < 0.01, ^***^p < 0.001, and n.s means no significant difference compared with the control group (Student’s *t*-test).

Increased expression of the cytokines IL-6, IL-10, and TNF-α is closely related with severe COVID-19. We sought to explore the relationship between increased expression of cytokines and ORF7b expression. The plasmid expressing flag-ORF7b was transfected into the HEK293T cells or Vero E6 cells for 18 h. Then, the expression of IFN-β, TNF-α, and IL-6 was measured by QRT-PCR, ELISA, and Western blotting. QRT-PCR results showed that mRNA expression levels of IFN-β and TNF-α in HEK293T and Vero E6 cells were upregulated after flag-ORF7b overexpressed (Figures 2A,B). Then, expression of IL-6, IFN-β, and TNF-α in cell supernatants was measured by ELISA. As we expected, with the overexpression of ORF7b in cells, expression of IL-6, IFN-β, and TNF-α in cell-culture supernatants also increased (Figures 2C,D). The protein levels of IL-6 and TNF-α were also measured in HEK293T cells and Vero E6 cells. Western blotting results showed that expressions of IL-6 and TNF-α were upregulated when flag-ORF7b exists (Figure 2E).

### ORF7b Activates the Type-I IFN Signaling Pathway

**Figure 3:**
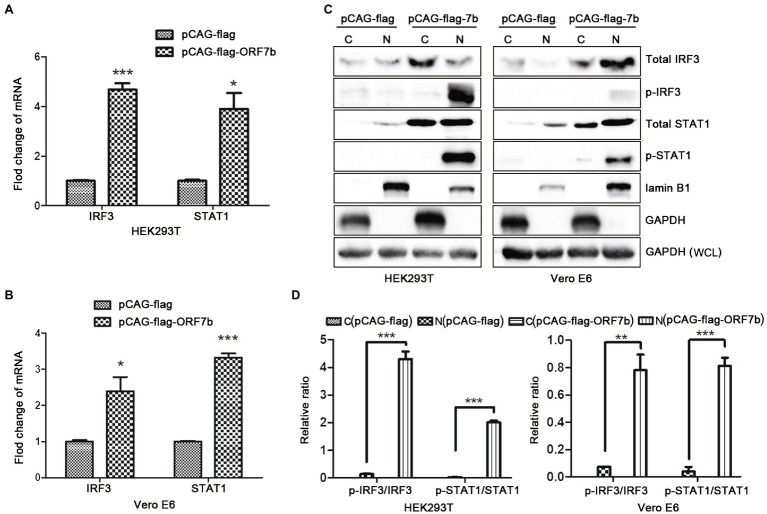
ORF7b activates the type-I interferon (IFN) signaling pathway. **(A)** mRNA expression of interferon regulatory transcription factor (IRF)-3 and signal transducer and activator of transcription (STAT)-1 in HEK 293 T cells were determined by QRT-PCR at 18 h after transfection with pCAG-flag and pCAG-flag-ORF7b. **(B)** mRNA expression of IRF3 and STAT1 in Vero E6 cells was determined by QRT-PCR at 18 h after transfection with pCAG-flag and pCAG-flag-ORF7b. **(C)** Protein expression of total STAT1, total IRF3, p-STAT1, and p-IRF3 in the cytoplasm (C) or nucleus (N) of Vero E6 cells and HEK293T cells was measured by Western blotting after transfection with pCAG-flag-ORF7b for 18 h. **(D)** Calculation of the expression of total STAT1,total IRF3, p-STAT1, and p-IRF3 in the cytoplasm or nucleus of Vero E6 cells and HEK293T cells. The quantification was finished by ImageJ for three times. Data are shown as mean ± SD, *n* = 3. ^*^p < 0.05; ^**^p < 0.01; ^***^p < 0.001, compared with the control group (Student’s *t*-test was used for analysis of A,B and one-way ANOVA Tukey’s *post-hoc* test was used for D).

IFN-β is an important type-I IFN. IFN-β is a glycoprotein secreted by fibroblasts and leukocytes under the stimulation of viruses. We discovered that IFN-β expression was upregulated upon ORF7b stimulation (Figure 2), so we further explored if ORF7b could activate the type-I IFN signaling pathway. Vector pCAG-flag-ORF7b was transfected transiently into cells for 18 h, and the expression of IRF3 and STAT1 (which are important transcription factors in the type-I IFN signaling pathway) was measured after overexpression of ORF7b. QRT-PCR results showed that transcription of *IRF3* and *STAT1* was upregulated in HEK293T cells and Vero E6 cells after ORF7b overexpression (Figures 3A,B). In addition, protein levels of total IRF3 and total STAT1 were also increased in ORF7b overexpression cells (Figure 3C). In order to investigate whether ORF7b promotes the phosphorylation and nuclear entry of these two key transcription factors or not. After flag-ORF7b expressed in the two cell lines, we extracted the cytoplasmic and nuclear proteins, respectively, and calculated the difference in expression of p-IRF3 and p-STAT1.Western blotting results showed that, compared with the control group, expression of p-STAT1 and p-IRF3 in nucleus of the ORF7b-transfected group was increased (Figure 3C), and the ratio of p-IRF3/total IRF3 and p-STAT1/total STAT1 were also increased (Figure 3D). Collectively, these data suggested ORF7b promoted the phosphorylation of IRF3/STAT1, and then activated the type-I IFN signaling pathway.

### ORF7b Mediates TNF-Induced Apoptosis

**Figure 4:**
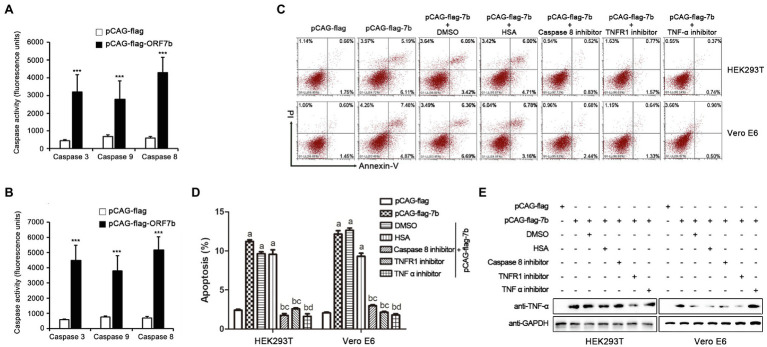
Caspase 8, TNFR1, and TNF-α inhibitor suppress ORF7b induced apoptosis. **(A)** Enzyme activity changes of caspase 3, 8, and 9 in HEK293T cells after transfection with pCAG-flag and pCAG-flag-ORF7b for 18 h. **(B)** Enzyme activity changes of caspase 3, 8, and 9 in Vero E6 cells after transfection with pCAG-flag and pCAG-flag-ORF7b for 18 h. **(C)** Apoptosis in HEK293T and Vero E6 cells was detected by flow cytometry after transfected with pCAG-flag/pCAG-flag-ORF7b or treatment with Caspase 8 inhibitor, TNFR1 inhibitor, and TNF-α inhibitor for 18 h. DMSO and HSA were set as the controls for Caspase8/TNFR1 inhibitor and TNF-α inhibitor groups, respectively. **(D)** The percentage of apoptosis was calculated from three independent experiments. a, vs. pCAG-flag group, ^***^; b, vs. pCAG-flag group, n.s; c, vs. pCAG-flag-ORF7b plus DMSO group, ^***^; and d, vs. pCAG-flag-ORF7b plus HSA group, ^***^. **(E)** The expression level of TNF-α was detected by Western blotting after treatment for 18 h. Data are shown as mean ± SD, *n* = 3. ^***^p < 0.001 and n.s, means no significant difference, compared with the relative control group. Student’s *t*-test was used for analysis of A,B; one-way ANOVA and Tukey’s *post-hoc* test were used for D.

According to the above results, ORF7b not only induced apoptosis, but also activated cells to overexpress TNF-α. Therefore, ORF7b may activate a TNF signaling pathway that would cause apoptosis. Then, the expression of caspase 3, caspase 8, and caspase 9 was measured at 18 h after pCAG-flag-ORF7b transfection. ORF7b highlighted the activity of caspases 3, 8, and 9 in HEK 293 T cells and Vero E6 cells (Figures 4A,B). While the inhibitors which function on caspase 8, type ITNF receptor (TNFR1) and TNF-α suppressed the cellular apoptosis caused by ORF7b in both HEK293T and Vero E6 cells (Figures 4C,D). The inhibitors also suppressed the expression of TNF-α in the two cell lines, especially targeting to the caspases 8 and TNFR1 (Figure 4E). Taken together, these results indicate that ORF7b activates cellular apoptosis *via* the TNF-α pathway.

**Figure 5:**
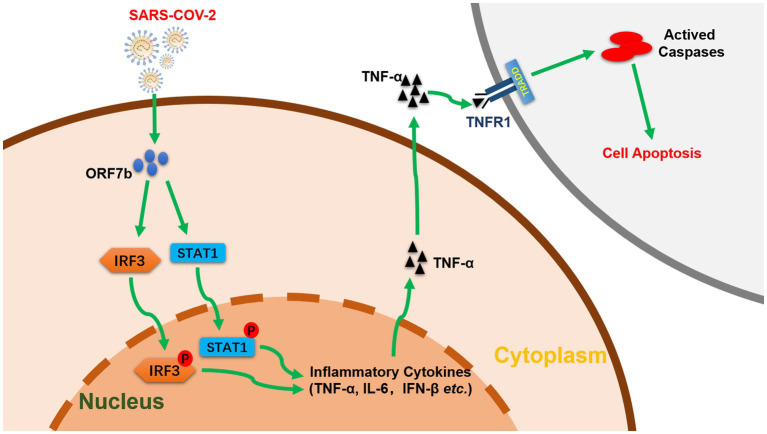
Function and pathway of ORF7b in cell apoptosis (schematic). ORF7b promotes the phosphorylation of IRF3 and STAT1, which translocate to the nucleus and activate TNF-α secretion, thereby resulting in cellular apoptosis through TNFR1 pathways.

In summary, we clarified that an accessory protein ORF7b of SARS-CoV-2 induces TNF-α/IL6/IFN-β expression and enhances phosphorylation of IRF3 and STAT1. We also demonstrate that ORF7b induces apoptosis through a TNF signaling pathway (Figure 5). These findings aid in understanding of the pathogenicity of SARS-CoV-2 and provide mechanistic insights in the interplay between SARS-CoV-2 infection and host innate immunity.

## Discussion

SARS-CoV-2 infection causes patients with severe COVID-19 to suffer lymphopenia and the cytokine storm. Accessory proteins of SARS-CoV-2 have important roles in regulating the host response to viral infection. So far, the function of accessory protein ORF7b of SARS-CoV-2 is largely unknown. ORF7b of SARS-CoV-2 shares 81.4% sequence similarity with that of the Urbani strain of SARS-CoV (GenBank: AY278741.1). ORF7b of SARS-CoV-2 contains a transmembrane region and is located in the membrane of the endoplasmic reticulum (Zhang et al., 2020), whereas for SARS-CoV, it is localized in Golgi apparatus (Schaecher et al., 2008), potentially having different functions. Remote homologues predicted by Galaxy WEB (A web server for protein structure prediction)^1^ showed the abundant sheet form a barrel and caused polymers, and whether it is polymerized to exert activity remains to be further studied.

Recent studies have shown that ORF7b of SARS-CoV-2 interacts with mitochondrial antiviral signaling protein (MAVS) and Unc-93 homolog B1 (UNC93B1; Stukalov et al., 2020), both of which have functions in innate and adaptive immunity. The adaptor protein MAVS activates the downstream TANK-binding kinase-1, which then phosphorylates the transcription factor IRF3. Subsequently, phosphorylated IRF3 dissociates from the adaptor protein and dimerizes, and then translocates into the nucleus to drive production of type-I IFN. STAT1 is recruited directly into the toll-like receptor (TLR) signaling pathway. p-STAT1 promotes translocation of itself to the nucleus and mediates the responses of specific pro-inflammatory cytokine following TLR stimulation (e.g., TNF-α and IL-6; Luu et al., 2014), which are the main components of the cytokine storm. We demonstrated that ORF7b promoted translocation of p-IRF3 and p-STAT1 from the cytoplasm to the nucleus. ORF7b promoting the nuclear translocation of IRF3 or STAT1 may be the result of ORF7b interacting with cellular MAVS or UNC93B1, but this hypothesis needs testing.

Recent studies have shown that SARS-CoV-2 can induce expression of multiple IFN-stimulated genes (Blanco-Melo et al., 2020; Zhou et al., 2020). ORF6 of SARS-CoV and SARS-CoV-2 antagonize IFN production and downstream signaling (Lei et al., 2020). In the present study, ORF7b of SARS-CoV-2 could activate an IFN signaling pathway, but we also revealed that ORF7b activated IFN-β signaling and induced apoptosis through TNFR1 and caspase 8. Binding of TNF-α to TNFR1 leads to the formation of death-inducing signaling complex and induces RIP1-dependent activation of caspase8 and apoptosis (Nikoletopoulou et al., 2013). ORF3a of SARS-CoV-2 also induces higher levels of apoptosis in Vero E6, HEK293T, and HepG2 cells *via* an extrinsic pathway involving caspases3, 8, and 9 (Ren et al., 2020) and, in the present study, ORF7b used a similar strategy to induce apoptosis. There may be a possibility that cell apoptosis is caused by forced expression of exogenous protein. However, it is unlikely to cause the phenotype we observed, as ORF7b induced apoptosis to a similar level as ORF3a.

Taken together, we investigated the interaction between ORF7b of SARS-CoV-2 and host antiviral responses. ORF7b may promote the type-I IFN signaling pathways and eventually accelerate TNF-induced apoptosis in HEK293T cells and Vero E6 cells. These findings could provide insights into the interactions between SARS-CoV-2 and the host, broaden our understanding of the pathogenicity of this deadly virus.

## Data Availability Statement

The raw data supporting the conclusions of this article will be made available by the authors, without undue reservation.

## Author Contributions

LL and ZL contributed to the design of experiments and writing of the manuscript. RY, QZ, JR, FZ, SY, MJ, and XS contributed to the conduction of experiments. RY, FZ, JR, JL, and SY contributed to reagent procurement. JY, MJ, ZJ, JC, XS, and ZJ contributed to data analyses. JY and LL contributed to the editing of the manuscript. All authors contributed to the article and approved the submitted version.

## Conflict of Interest

The authors declare that the research was conducted in the absence of any commercial or financial relationships that could be construed as a potential conflict of interest.

## Publisher’s Note

All claims expressed in this article are solely those of the authors and do not necessarily represent those of their affiliated organizations, or those of the publisher, the editors and the reviewers. Any product that may be evaluated in this article, or claim that may be made by its manufacturer, is not guaranteed or endorsed by the publisher.

## Footnotes

^1^ http://galaxy.seoklab.org/
