# Supplementary material for: SARS-CoV-2 Accessory Protein ORF7b Mediates Tumor Necrosis Factor-α-Induced Apoptosis in Cells
Source: Front Microbiol. 2021 Aug 13;12:654709. doi: 10.3389/fmicb.2021.654709 (PMC8414645; doi:10.3389/fmicb.2021.654709)
Supplement: Supplementary file 1 [file Table_1.docx]

Table S1. Primer for Quantitative PCR.

| Nucleic acid name | Sequence, 5’-3’ |
| --- | --- |
| *TNFα*-F | GTCAACCTCCTCTCTGCCAT |
| *TNFα*-R | CCAAAGTAGACCTGCCCAGA |
| *IFNβ*-F | TCTCCTGTTGTGCTTCTCCA |
| *IFNβ*-R | TCAATTGCCACAGGAGCTTC |
| *IL6*-F | AATTCGGTACATCCTCGACGG |
| *IL6*-R | GGTTGTTTTCTGCCAGTGCC |
| *IRF3*-F | CCGCCAAGTCTTCCAGCAG |
| *IRF3-*R | TGCCTCACGTAGCTCATCA |
| *STAT1*-F | GAGAGTCTGCAGCAAGTTCG |
| *STAT1*-R | TTTCCACCACAAACGAGCTC |
| *GAPDH*-F | TGGAAGGACTCATGACCACA |
| *GAPDH*-R | AGGCAGGGATGATGTTCTGG |

F, forward; R, reverse;
